# Exploring the Diversity of Fungal DyPs in Mangrove Soils to Produce and Characterize Novel Biocatalysts

**DOI:** 10.3390/jof7050321

**Published:** 2021-04-21

**Authors:** Amal Ben Ayed, Geoffroy Saint-Genis, Laurent Vallon, Dolores Linde, Annick Turbé-Doan, Mireille Haon, Marianne Daou, Emmanuel Bertrand, Craig B. Faulds, Giuliano Sciara, Martino Adamo, Roland Marmeisse, Sophie Comtet-Marre, Pierre Peyret, Danis Abrouk, Francisco J. Ruiz-Dueñas, Cyril Marchand, Mylène Hugoni, Patricia Luis, Tahar Mechichi, Eric Record

**Affiliations:** 1 INRAE, UMR1163, Biodiversité et Biotechnologie Fongiques, Aix-Marseille Université, 13288 Marseille, France; amal.benayed@enis.tn (A.B.A.); annick.doan@univ-amu.fr (A.T.-D.); mireille.haon@inrae.fr (M.H.); mariane.daou@ku.ac.ae (M.D.); emmanuel.bertrand@univ-amu.fr (E.B.); craig.faulds@univ-amu.fr (C.B.F.); giuliano.sciara@inrae.fr (G.S.); 2 Laboratoire de Biochimie et de Génie, Enzymatique des Lipases, Université de Sfax, Ecole Nationale d’Ingénieurs de Sfax, 3038 Sfax, Tunisia; tahar.mechichi@enis.rnu.tn; 3 Université Lyon, Université Claude Bernard Lyon 1, CNRS, INRAE, VetAgro Sup, UMR Ecologie Microbienne, 69622 Villeurbanne, France; geoffroy.saint-denis@univ-lyon1.fr (G.S.-G.); laurent.valon@univ-lyon1.fr (L.V.); martino.adamo@univ-lyon1.fr (M.A.); patricia.luis@univ-lyon1.fr (P.L.); roland.marmeisse@univ-lyon1.fr (R.M.); danis.abrouk@univ-lyon1.fr (D.A.); mylene.hugoni@univ-lyon1.fr (M.H.); 4 Centro de Investigaciones Biológicas Margarita Salas (CIB), CSIC, 28040 Madrid, Spain; lolalinde@cib.csic.es (D.L.); fjruiz@cib.csic.es (F.J.R.-D.); 5 Dipartimento di Scienze della Vita e Biologia dei Sistemi, Università degli Studi di Torino, 10125 Torino, Italy; 6 Université Clermont Auvergne, INRAE, MEDiS, 63000 Clermont-Ferrand, France; sophie.comtet-marre@inrae.fr (S.C.-M.); pierre.peyret@inrae.fr (P.P.); 7 IMPMC, Institut de Recherche Pour le Développement (IRD), UPMC, CNRS, MNHN, 98851 Noumea, France; cyril.marchand@unc.nc; 8 ISEA, EA, Université de la Nouvelle-Calédonie (UNC), 3325, BP R4, 98851 Noumea, France; 9 Department of Chemistry, Khalifa University, P.O. Box 127788, Abu Dhabi, United Arab Emirates

**Keywords:** lignocellulose degrading enzymes, dye-decolorizing peroxidases, heterologous expression, dye decolorization, marine fungus, mangrove, salt adaptation

## Abstract

The functional diversity of the New Caledonian mangrove sediments was examined, observing the distribution of fungal dye-decolorizing peroxidases (DyPs), together with the complete biochemical characterization of the main DyP. Using a functional metabarcoding approach, the diversity of expressed genes encoding fungal DyPs was investigated in surface and deeper sediments, collected beneath either *Avicennia marina* or *Rhizophora stylosa* trees, during either the wet or the dry seasons. The highest DyP diversity was observed in surface sediments beneath the *R. stylosa* area during the wet season, and one particular operational functional unit (OFU1) was detected as the most abundant DyP isoform. This OFU was found in all sediment samples, representing 51–100% of the total DyP-encoding sequences in 70% of the samples. The complete cDNA sequence corresponding to this abundant DyP (OFU 1) was retrieved by gene capture, cloned, and heterologously expressed in *Pichia pastoris*. The recombinant enzyme, called DyP1, was purified and characterized, leading to the description of its physical–chemical properties, its ability to oxidize diverse phenolic substrates, and its potential to decolorize textile dyes; DyP1 was more active at low pH, though moderately stable over a wide pH range. The enzyme was very stable at temperatures up to 50 °C, retaining 60% activity after 180 min incubation. Its ability to decolorize industrial dyes was also tested on Reactive Blue 19, Acid Black, Disperse Blue 79, and Reactive Black 5. The effect of hydrogen peroxide and sea salt on DyP1 activity was studied and compared to what is reported for previously characterized enzymes from terrestrial and marine-derived fungi.

## 1. Introduction

Coastal mangrove soils are fascinating ecosystems, representing a whole forest environment at the interface between land and sea. These ecosystems consist mainly of woody plants that grow under extreme environmental conditions such as high salinity and high temperature [1]. Mangrove leaves and sediments contain a high concentration of carbonaceous material, feeding a considerable bacterial and fungal microflora [2,3]. According to Latha [4], mangrove fungi represent the second largest ecological group of all marine fungi. Recently, the structure of microbial communities in New Caledonian mangrove sediments was analyzed in detail using a metabarcoding approach [3]. This work investigated the distribution of prokaryotic and fungal communities with respect to depth, vegetation cover, and season. The prokaryotic community appeared to be exclusively shaped by sediment depth, with resulting differences in prokaryotic phyla composition. On the contrary, the fungal community was evenly distributed according to the above criteria and showed a dominance of Ascomycota over Basidiomycota in all analyzed layers [3]. Mangroves are considered to be the largest carbon reservoir in coastal ecosystems and actively supply carbon to adjacent ecosystems [5,6]. In this carbon-rich environment, mangrove fungi play a key role in the recycling of organic matter, including Lignocellulose-rich biomass [7]. In terrestrial forests, fungal degradation of plant lignocellulose is based on a close association with the woody material, and on the secretion of a complex and variable cocktail of enzymes acting in a sequential, sometimes synergistic way [8,9]. Lignocellulolytic enzymes, including cellulases, hemicellulases, and lignin-degrading enzymes (including Ligninolytic peroxidases and laccases), are classified in the CAZy database [10]. The scientific literature related to their characterization is broad and diverse for enzymes derived from terrestrial species. By contrast, very little is known about the enzymatic mechanisms employed by mangrove-derived fungi to break down plant biomass and their adaptation to marine conditions, especially to high salt concentrations. In a previous study, we showed that the presence of sea salt modified the composition of secreted lignocellulolytic enzymes, with increased secretion of xylanases and cellulases, and lower production of oxidoreductases belonging to the auxiliary activities (AA) class of the CAZy database [11]. This tendency was recently confirmed in the study of the marine-derived fungus *Stemphylium lucomagnoense* [12]. Although terrestrial basidiomycetes, particularly white-rot fungi, are seen as the key actors in environmental lignin degradation, marine-derived ascomycetes were also demonstrated to significantly degrade lignin [13]. For example, the mangrove fungus *Pestalotiopsis* sp. was demonstrated to produce two different laccases that are active, with different enzymatic behavior, in up to 5% sea salt [14]. Similarly, the marine white-rot basidiomycete *Phlebia* sp. was shown to secrete two enzymatically different manganese peroxidases (MnPs), in saline and non-saline conditions, respectively, also illustrating the adaptation of marine fungi to sea salt [15].

Classical ligninolytic heme peroxidases, including manganese peroxidases (MnPs), lignin peroxidases (LiPs), and versatile peroxidases (VPs), belong to the peroxidase-catalase superfamily [16]. Dye-decolorizing peroxidases (DyPs) are heme peroxidases that were more recently described. They belong, together with chloride dismutases (Cld) and other heme-binding proteins (EfeB gene), to the “dimeric α + β barrel structural superfamily” (Pfam CL0032, SCOP identifier 54909), also called CDE superfamily [17,18]. However, the evolutionary relationships among the CDE family members are not clear: they show low identity in structure-based alignments, and their common folding could represent a convergent or divergent evolutionary process [19]. The fold of these proteins consists of a β barrel decorated with α helices, resulting from homo- or hetero-dimerization of two ferredoxin-like motifs [20]. Each motif is supplied by distinct polypeptide chains, like in some cofactor-free bacterial enzymes [21,22], or by the N and C terminal domains of a single polypeptide, like in DyPs, each containing a conserved histidine in the heme-binding site and a GXXDG signature motif. This sequence contains the catalytic aspartate that acts as a proton acceptor, playing the role of the catalytic histidine found in plant peroxidases [23,24]. DyPs are produced in bacteria [25] as well as in filamentous fungi, with many examples in basidiomycetes such as *Bjerkandera adusta* [26], *Pleurotus ostreatus* [27], *Auricularia auricula-judae* [28,29], and *Trametes versicolor* [30]. They have been purified and biochemically characterized, demonstrating their capacities to oxidize a large variety of substrates, such as phenolic compounds (2, 6-dimethoxyphenol and guaiacol) and non-phenolic compounds (veratryl alcohol and Mn^2+^), together with anthraquinone substrates and also flavonoids extracted from oak wood (catechin and quercetin) [30,31]. While heme peroxidases have been largely studied in fungi isolated from terrestrial environments, little is known about their involvement in lignocellulose degradation in marine habitats. In comparison, other ligninolytic enzymes, such as laccases isolated from marine-derived fungi, have already been isolated, produced, and characterized [32,33], and some have been shown to participate in lignocellulose breakdown [34] or be promising candidates for biotechnological processes such as dye decolorization [33,35].

In the present work, a cDNA capture by hybridization approach was used (i) to evaluate the diversity of expressed fungal genes that encode DyPs in mangrove sediments, using a metabarcoding approach, and (ii) to recover and clone full-length DyP cDNAs in *P. pastoris*, to gain insights into the biochemical properties of enzymes isolated from marine environments. One DyP, called DyP1 in this study, was heterologously expressed and characterized, and its biotechnological potential for dye decolorization was assessed.

## 2. Materials and Methods

### 2.1. Strains for Cloning and Heterologous Expression

*Escherichia coli* strain DH5α (Promega, Charbonnieres, France) was used for vector storage and propagation. *P. pastoris* strain X33 (Invitrogen, Carlsbad, CA, USA) was used for the heterologous expression of the DyP-encoding synthetic cDNA after optimization of codons (GenScript, Piscataway, NJ, USA).

### 2.2. Sediment Sampling, RNA Extraction, and cDNA Synthesis

Sediment samples were collected from a mangrove wetland located in Saint Vincent Bay (21°55′58′′ S, 166°4′30′′ E) on the west coast of New Caledonia. As previously described [3], sediment samples were collected in three independent 10 m^2^ plots (A, B, and C) located 50 m apart and defined in *Avicennia marina* (A) and *Rhizophora stylosa* (R) pristine areas. Three sediment cores (50 cm deep) were collected in 2016 at low tide with a stainless-steel corer (8 cm diameter) in each 10 m^2^ plot during the wet (March) and dry (November) seasons. Oxic (0–10 cm deep) and anoxic (40–50 cm deep) fractions were collected from each core, and a single composite sample per fraction and per plot (A, B, or C) for each tree area (*A. marina* and *R. stylosa*) was prepared by mixing equal amounts of sediments. Per season (March or November), a total of 12 different composite samples were thus obtained and separately analyzed: (i) R1A, R1B, and R1C and R2A, R2B, and R2C corresponding, respectively, to the oxic and anoxic fractions from the three plots (A, B, and C) designated in the *R. stylosa* area (R). (ii) A1A, A1B, and A1C and A2A, A2B, and A2C corresponding, respectively, to oxic and anoxic fractions of the three plots (A, B, or C) localized in the *A. marina* area (A). All composite samples were frozen and kept at −70 °C until use.

Total RNA was extracted from 8–12 g of each composite sample using the RNeasy PowerSoil Total RNA kit according to the manufacturer’s recommendations (Qiagen, Germantown, MD, USA). RNA quality was evaluated on 1% agarose gels and the absence of DNA contamination confirmed by PCR using non-reverse transcribed mRNA and eukaryotic constitutively expressed EF1α gene-specific primers [36]. Specific reverse transcription of poly-A mRNA, followed by double-stranded cDNA synthesis and amplification, was performed on 500 ng of total RNA using the Mint-2 cDNA synthesis kit (Evrogen, Moscow, Russia). The optimal number of PCR cycles to maintain a balance between transcript representation and nonspecific background amplification during the cDNA amplification was estimated to be 27 cycles. The resulting cDNAs, previously purified using a phenol-chloroform protocol [37], were used as templates to specifically capture by hybridization and sequence expressed fungal genes encoding DyPs.

### 2.3. Probe Design, cDNA Capture by Hybridization, and High-Throughput Sequencing of Fungal DyP cDNA

First, 1267 publicly available fungal DyP DNA coding sequences were identified by BLAST searches and collected from GenBank (http://www.ncbi.nlm.nih.gov/genbank/, accessed on 19 April 2021), the Joint Genome Institute Mycocosm database (https://mycocosm.jgi.doe.gov/mycocosm/home, http://www.ncbi.nlm.nih.gov/genbank/, accessed on 19 April 2021; [38]) and the specialized RedOxiBase (http://peroxibase.toulouse.inra.fr/, accessed on 19 April 2021; [39]). This sequence data set was used to design 69 (70 bp long) degenerate capture probes using the KASpOD software (https://g2im.u-clermont1.fr/kaspod/index.php, accessed on 19 April 2021); [40] (individual probe sequences will be published in a separate paper on fungal DyP diversity). In silico, individual probes could hybridize to 0.2–11% of the 1267 fungal DyP sequences (four allowed mismatches). Oligonucleotides were synthesized as single-strand DNA flanked by two adaptor sequences (ATCGCACCAGCGTGT and CACTGCGGCTCCTCA) for their PCR amplification and conversion to biotinylated RNA probes using the T7 RNA polymerase.

cDNA capture by hybridization was carried out as described by Bragalini et al. (2014) [41]. Briefly, 2 μg of heat-denatured PCR-amplified cDNAs was hybridized to the equimolar mix of biotinylated RNA probes (500 ng) for 24 h at 65 °C. Probe/cDNA hybrids were captured on streptavidin-coated paramagnetic beads (Dynabeads^®^ M-280 Streptavidin, Invitrogen). After different washing steps to remove unbound cDNAs, the captured cDNAs were eluted using 50 μL of 0.1 M NaOH at room temperature, neutralized with 70 μL of 1 M Tris-HCl (pH 7.5), and purified using the MinElute PCR purification kit (Qiagen). Captured cDNAs were amplified using the primer M1 (Mint-2 cDNA synthesis kit, Evrogen) that binds at both 5′ and 3′ ends of the cDNAs. PCR amplifications were performed in 50 μL reaction mixtures containing 5 μL of captured cDNA, 200 μM of dNTPs, 400 nM of primer M1, 5 μL of 10X Encyclo buffer, and 1 μL of 50X Encyclo DNA polymerase (Evrogen). Cycling conditions were 1 min at 95 °C followed by 25 cycles of 15 s at 95 °C, 20 s at 66 °C, and 3 min at 72 °C. Ten independent amplifications were conducted for each sample. PCR products of the same sample were purified using the MinElute PCR purification kit (Qiagen, Courtaboeuf, France) and pooled. A second round of hybridization and PCR amplification was performed using each of the amplified cDNA samples obtained after the first hybridization capture.

Captured cDNAs of each sediment sample (12 per season) were used as templates to specifically amplify fragments of expressed fungal DyP genes using the following fungal-specific tagged degenerate primers: DyP-F, 5′-*Tag*-TGYCCITTYGCIGCNCAYAT-3′ and DyP-R, 5′-*Tag*-RAARAARTAYTCICCNCC-3′ (Table S1; [42]. All PCR amplifications were performed in triplicates in 25 μL reaction mixtures containing 20 ng of amplified cDNA, 2.5 μL of 10X polymerase buffer (Invitrogen), 0.75 μL of MgCl_2_ (50 mM), 2.5 μL of dNTPs (2 mM each), 1 μL of each primer (20 μM, Invitrogen), 0.3 μL of BSA (20 mg mL^−1^), and 0.1 μL of *Taq* DNA polymerase (5U·μL^−1^, Invitrogen). Cycling conditions were 3 min at 94 °C and 35 cycles of 45 s at 94 °C, 45 s at 50 °C, and 45 s at 72 °C, followed by 10 min at 72 °C. Control reactions without nucleic acid were systematically run in parallel. Amplicons from the three independent PCR reactions were pooled and purified using the Agencourt AMPure XP Kit (Beckman Coulter Diagnostics, California, CA, USA) and quantified by fluorometry using a Qubit 2.0 fluorimeter and the Qubit dsDNA HS assay kit (Invitrogen, ThermoFisher Scientific, Waltham, MA, USA). Per season (March or November), an equimolar mix of the tagged PCR products obtained for the 12 different sediment samples was prepared and sequenced by FASTERIS (FASTERIS, Plan-les-Ouates, Switzerland) on an Illumina MiSeq sequencer (2 × 250 bp).

In parallel, four full-length-captured cDNA composite samples corresponding to the oxic and anoxic fractions collected in the *A. marina* (A1, A2) and *R. stylosa* (R1, R2) areas were generated by pooling an equal amount of captured cDNAs obtained for the different sediment samples of each season. These four full-length captured cDNA samples were sequenced using the Illumina HiSeq 2000 2 × 250 bp technology (I.G.A. Technologies, Udine, Italy).

### 2.4. Bioinformatic Analysis and Statistics

Concerning the Miseq raw data, fungal DyP paired-end reads were merged using Pear [43] and demultiplexed. Denoising procedures consisted of discarding reads that fell outside the expected length range (expected size 350–470 bp) and those containing ambiguous bases (N). Sequences were clustered into operational functional units (OFUs) using SWARM [44]. SWARM is a de novo clustering based on an unsupervised single-linkage clustering method that reduces the impact of clustering parameters on the resulting OFUs by avoiding arbitrary global clustering thresholds and input sequence ordering dependencies. SWARM builds OFUs in two steps: (i) an initial set of OFUs was constructed by iteratively agglomerating similar amplicons, and (ii) amplicon abundance values were used to reveal OFUs internal structures and to break them up into sub-OFUs if necessary. In the present work, the SWARM aggregation distance equaled to 3. Chimeras were removed using VSEARCH [45], and low abundance sequences accounting for less than 0.005% of the dataset were filtered out. This whole procedure was performed using the pipeline FROGS [46]. To be able to compare samples, a normalization procedure was applied to randomly resample down to 32,556 sequences per sample. A Wilcoxon test was used to statistically evaluate the differences between the alpha diversity indices calculated (Shannon index (H’), complement of the Simpson index (1-D), and Evenness (J’)). The effect of environmental factors (tree, season, and depth) on the composition of expressed fungal genes encoding DyPs were tested using nonparametric permutation-based multivariate analysis of variance (PERMANOVA, adonis function; [47] based on abundance dissimilarity (Bray–Curtis) matrices). These analyses were performed using the VEGAN package (http://cran.r-project.org, accessed on 19 April 2021) in R. The sequence data generated in this study were deposited in the EMBL-ENA public database (PRJEB43346 for the first sampling campaign (March) dataset and PRJEB43343 for the second sampling campaign (November) dataset).

Concerning the HiSeq raw data for full-length cDNA reconstruction through gene capture by hybridization enrichment, adapter sequences were eliminated using Cutadapt [48]. Sequence quality was evaluated with Trimmomatic [49]; bases with quality lower than 20 were eliminated and only sequences longer than 60 bases were kept. Trimmed sequences were then assembled using IDBA-UD [50] (default parameters) and resulting contigs were further assembled with CAP3 [51] (default parameters) to obtain longer contigs. A similarity search between a custom peroxidase database and contigs was performed using DIAMOND [52], with the BLASTx command in “sensitive” mode (i.e., a maximal *E*-value of 1 × 10^−5^ and a minimal identity of 50%). Matching sequences were further analyzed to search the DYP peroxidase domain using ScanProSite [53] and the Prosite database [54].

The ProtParam tool (http://web.expasy.org/protparam/, accessed on 19 April 2021) was used to predict the theoretical pI, molecular mass, and molar extinction coefficient of DyP. For sequence comparison, BlastP was used to search for sequences with similarity to DyP1 in the UniProtKB/Swiss-Prot database (http://www.uniprot.org/blast, accessed on 19 April 2021). The search parameters were scoring matrix BLOSUM 62, gapped alignment allowed, and cut-off *E*-value 0.1. DyP1 was aligned with 241 DyP sequences identified in Agaricomycotina genomes available at the MycoCosm portal (https://mycocosm.jgi.doe.gov/mycocosm/home, accessed on 19 April 2021) and GenBank using MUSCLE as implemented in MEGA X (https://www.megasoftware.net/, accessed on 19 April 2021) [55]. A maximum likelihood phylogenetic tree was then constructed by MEGA X using the WAG evolutionary model with gamma-distributed rate variation and the amino acid frequencies of the dataset.

### 2.5. Cloning and Expression of DyP-encoding cDNA

DyP1 was produced using the in-house 3PE Platform (*P. pastoris* Protein Express: www.platform3pe.com/, accessed on 19 April 2021). The cDNA encoding DyP was synthesized after codon optimization for *P. pastoris* (GeneArt, Regensburg, Germany) and inserted into the vector pPICZαA (Invitrogen, Cergy-Pontoise, France) using *Xho*I and *Xba*I restriction sites in frame adding a C-terminal (His)6-tag to the recombinant protein.

*P. pastoris* strain X33 and the pPICZαA vector are components of the *P. pastoris* Easy Select Expression System (Invitrogen). The *Pme*I-linearized pPICZαA recombinant plasmid was inserted into *P. pastoris* competent cells by electroporation. Zeocin-resistant transformants were then screened for protein production.

### 2.6. Production and Purification of Recombinant DyP

The best producing transformant was grown in 2.5 L of BMGY (10 g L^−1^ glycerol, 10 g L^−1^ yeast extract, 20 g L^−1^ peptone, 3.4 g L^−1^ YNB, 10 g L^−1^ ammonium sulfate, 100 mM phosphate buffer pH 6, and 0.2 g L^−1^ of biotin) in flasks shaken at 30 °C in an orbital shaker (200 rpm) for 16 h to an OD600 of 2–6. Expression was induced by transferring the cells into 500 mL of BMMY (10 g L^−1^ yeast extract, 20 g L^−1^ peptone, 3.4 g L^−1^ YNB, 10 g L^−1^ ammonium sulfate, 100 mM phosphate buffer pH 6, and 0.2 g L^−1^ of biotin) adding 0.1 or 0.5 gL^−1^ of hemin at 20 °C in an orbital shaker (200 rpm) for a further three days. Each day the medium was supplemented with 3% (*v/v*) methanol.

The supernatant was collected after harvesting cells by centrifugation at 3500× *g* for 5 min at 4 °C. After adjusting the pH to 7.8, the supernatant was filtered on 0.45 µm filters (Millipore, Molsheim, France) and loaded onto 5 mL HisTrap HP columns (GE healthcare, Buc, France) connected to an Akta Xpress system (GE Healthcare). Prior to loading, the column was equilibrated with buffer A Tris-HCl 50 mM pH 7.8, NaCl 150 mM, and imidazole 10 mM. The (His)6-tagged recombinant enzyme was eluted with buffer B Tris-HCl 50 mM pH 7.8, NaCl 150 mM, and imidazole 500 mM. The fractions eluted containing the purified protein were pooled, concentrated with a 10 kDa vivaspin concentrator unit (Sartorius, Plaiseau, France), and dialyzed against 50 mM sodium acetate buffer pH 5.2.

Protein concentration was determined using a Nanodrop ND-2000 spectrophotometer (Thermo Fisher Scientific, IL, USA) by adsorption at 280 nm with theoretical molecular masses and molar extinction coefficients calculated from protein sequence using Expasy tools. A fraction of eluate was loaded onto 10% Tris-glycine precast SDS-PAGE (Bio-Rad, Marnes-la-Coquette, France) to check protein purity and integrity. The molecular mass under denaturing conditions was determined with PageRuler Prestained Protein Ladder (Thermo Fisher Scientific, IL, USA)

### 2.7. Structural Analysis

A 3D model of the DyP1 was obtained from the automated protein structure homology-modeling server SWISS-MODEL [56] using the crystal structure of *B. adusta* DyP (PDB 3MM3) as a template. The electrostatic surface was computed using the default parameters in PyMol. The putative glycosylation sites were predicted using NetOGlyc 4.0 Server [57].

### 2.8. Standard Conditions for Peroxidase Activity

DyP activity was estimated from the absorbance changes observed during substrate oxidation at optimal pH values at 30 °C in a Uvikon XS spectrophotometer (BioTek Instruments, Colmar, France) [29]. Hydrogen peroxide (0.25 mM) was added to initiate the reaction. Oxidation of 2,2′-azino-bis(3-ethylbenzothiazoline-6-sulfonic acid (ABTS) was followed by generation of its cation radical (ε_436_ = 29.3 mM^−1^ cm^−1^). RB19 oxidation was monitored for colorant disappearance (ε_595_ = 10 mM^−1^ cm^−1^). Substrate oxidation was determined by measuring the enzymatic activity using saturating concentrations of RB19 (600 µM) and ABTS (5 mM) in 100 mM of citrate–phosphate buffer.

### 2.9. Influence of Temperature and pH on DyP Activity and Enzyme Stability

To determine optimal temperature, the purified DyP was assayed over the temperature range of 20–70 °C in standard conditions. For the pH profiles, DyP activity was determined in 100 mM of citrate–phosphate buffer range from pH 2.6 to 7 using ABTS and RB19 as substrates at 30 °C.

To define the thermal stability, DyP aliquots were incubated at different temperatures (30–70 °C) for 30, 60, 90, 120, and 180 min. Thermal inactivation was stopped by immediately cooling the treated protein aliquot on ice, and activity was measured under standard conditions. The pH stability was determined by incubating DyP in 10 mM citrate-phosphate buffer at different pH (2.6, 3, 4, 5, and 6) for 4, 24, and 48 h at 30 °C, and then assaying the activity in standard conditions for each substrate.

### 2.10. Effect of Hydrogen Peroxide and Sea Salt on DyP Activity

The effects of H_2_O_2_ on peroxidase activity were determined under standard assay conditions at the optimal pH in the range of 0.1 to 5 mM in 0.1 M citrate–phosphate buffer at 30 °C.

The influence of sea salt on DyP activity was measured spectrophotometrically in standard assay conditions, as described above, after sea salt addition (1–5% wt/vol) using DyP of *Trametes versicolor* as a control [30].

### 2.11. Substrate Specificity and Kinetics

To determine the best substrates of the enzyme, enzymatic activities were measured using a UVIKONxs spectrophotometer (Bio-TEK Instruments) at optimal pH at 30 °C by following the oxidation of different substrates. For ABTS, 2,6-dimethoxyphenol (DMP) and veratryl acohol (VA) oxidation, absorbance increases at 436 (radical cation; ε_436_ = 29.3 mM^−1^ cm^−1^), 496 (dimeric coerulignone; ε_469_ = 55 mM^−1^ cm^−1^), and 310 nm (veratraldehyde; ε_310_ = 9.3 mM^−1^ cm^−1^) were followed, respectively. Absorbance decreases were followed in the case of Reactive Blue 19 (RB19) (595 nm, ε_595_ = 10 mM^−1^ cm^−1^) oxidation resulting in dye decolorization. The oxidation of Mn^2+^ was determined at 238 nm (Mn^3+^-tartrate complex; ε_238_ = 6.5 mM^−1^ cm^−1^) in 100 mM tartrate buffer pH 4.

All enzymatic activities were measured in linear increments (decreases for RB19). The Michaelis constant, *K*_m_, together with the enzyme turnover value, *k*_cat_, were obtained by non-linear least squares fitting of the experimental measurements to the Michaelis–Menten model. Fitting of these constants to the normalized equation *v* = (*k*_cat_/*K*_m_) [*S*]/(1 + [*S*]/*K*_m_) yielded the catalytic efficiency values (*k*_cat_/*K*_m_) with their corresponding standard errors.

### 2.12. Decolorization Properties

Five synthetic dyes, Acid Black (AB) (560 nm; 0.005% vol/vol), RB5 (610 nm; 0.0025% vol/vol), Disperse Blue 79 (DB79) (530 nm; 0.0005% vol/vol), Basic Blue 41 (BB41) (610 nm; 0.00001% vol/vol), and Vat Green (VG) (640 nm; 0.00025% vol/vol), were supplied by SETAS (Çerkezköy, Turkey) and used for determining the decolorization properties of DyP at 37 °C. The reaction mixture contained DyP (0.125 mg mL^−1^), dye solutions (final concentration described above), citrate–phosphate buffer (100 mM, pH 3), and 0.25 mM of H_2_O_2_ in a total volume of 1 mL. The enzymatic dye decolorization was detected by measuring the decrease in color absorbance in 1 h. The percentage of decolorization efficiency was calculated as follows:

Decolorization (%) = ((*A*_i_ − *A_t_*)/*A*_i_) × 100 (1)

where *A*_i_ is the initial absorbance of a dye, and *A_t_* is the absorbance of the dye after each time point *t*.

## 3. Results

### 3.1. Diversity and Capture of Fungal DyP Encoding cDNAs

**Figure 1:**
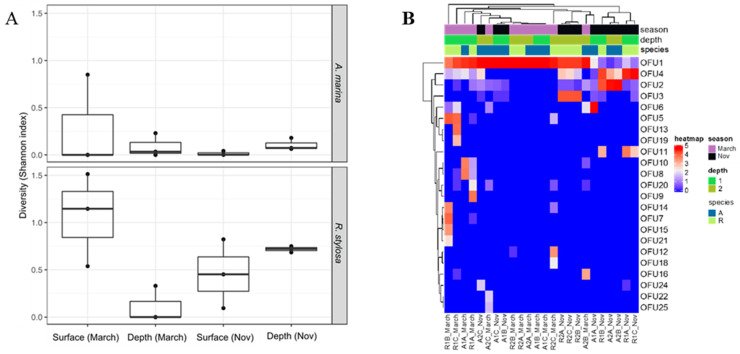
Diversity of expressed genes encoding fungal DyPs in surface and deeper sediments collected beneath two tree species (*Avicennia marina* and *Rhizophora stylosa*) during the wet (March) and dry (November) seasons. (**A**) Diversity estimated with the Shannon index and (**B**) distribution of the different Operational Functional Units (OFUs).

Regarding the distribution of OFUs, the most abundant one (OFU1) was detected in all sediment samples and represented 51,100% of all sequences in 70% of the samples (Figure 1B). The full-length cDNA sequence of OFU1 was obtained from the HiSeq dataset after gene capture by hybridization enrichment. The difference in the composition of expressed DyP-genes assessed through a nonparametric multivariate analysis of variance (PERMANOVA) highlighted a strong seasonal effect (P = 5.8 × 10^−5^, R^2^ = 0.178; Table 1). Tree species (P = 0.032, R^2^ = 0.062) and sediment depth (P = 0.021, R^2^ = 0.072) had a lower impact on the composition of the expressed fungal genes (Table 1).

The diversity of expressed genes encoding fungal DyPs was investigated in surface and deeper mangrove sediments beneath *A. marina* and *R. stylosa* trees during the wet (March) and dry (November) seasons. Sediment samples were collected in three independent plots (A, B, and C) in the *A. marina* (A) and *R. stylosa* (R) pristine areas. This expressed gene diversity was evaluated using a metabarcoding approach (Illumina Miseq) on environmental cDNAs previously enriched in DyP sequences by gene capture by hybridization. The normalized DyP dataset consisted of 781,344 sequences distributed among 25 different operational functional units (OFUs), corresponding to 25 putative DyP encoding cDNAs (Table S2). The number of OFUs per sediment sample varied from one to 10. DyP diversity, estimated with the Shannon index, was systematically higher in the surface layers during the wet season (Figure 1; Supplementary Table S2). The highest DyP diversity was observed for the surface layers beneath the *R. stylosa* area during the wet season. By contrast, the DyP diversity was systematically lower in the surface layers during the dry season (Figure 1A; Table S3).

### 3.2. Phylogenetic Analysis

We decided to study the most widely represented peroxidase in mangrove soils, OFU1, which we called DyP1. A BlastP search was conducted against the UniProtKB/Swiss-Prot database using DyP1 as the query. As a result, DyP sequences from fungal species of the orders Auriculariales, Sebacinales, and Geastrales, included in the subphylum Agaricomycotina, emerged as the proteins with the highest amino acid sequence identities to DyP1 (Table 2).

**Figure 2:**
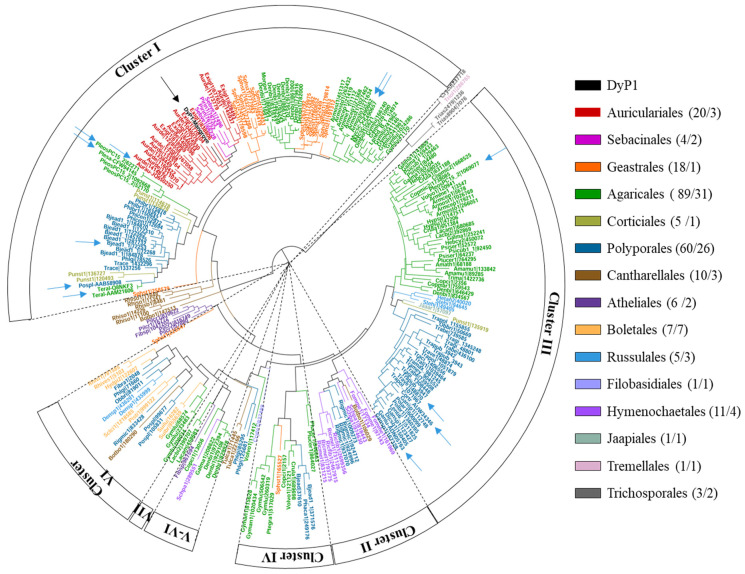
Maximum likelihood phylogenetic tree, constructed with 1000 bootstrap replications, showing the position of DyP1 from mangrove (black arrow) with respect to 233 DyP sequences identified in 82 published (and a few unpublished) Agaricomycotina genomes (the latter with permission of the PIs project) available at the JGI Mycocosm Portal on May 2020, including sequences of *Pleurotus ostreatus* (JGI 1069077 and JGI 62271), *Bjerkandera adusta* (JGI 72253), and *Trametes versicolor* (JGI 48870). Eight GenBank sequences from *Auricularia auricula-judae* (Aurau-JQ650250), *Mycetinis scorodonius* (Mycsc-CS490657 and Mycsc-CS490662), *Ganoderma lucidum* (Ganlu-ADN05763), *Polyporaceae* sp. (Pospl-AAB58908), *Termitomyces albuminosus* (Teral-AAM21606), *Pleurotus sapidus* (Plesa-CFW94145), and *Coriolopsis trogii* (Cortr_AUW34346) are also included. The color of both branches and enzymes indicates the order of the fungal species they belong to according to the legend, where the total DyP cDNA number is shown (including eight from GenBank) followed by the number of genomes for each order. The positions of the characterized DyPs from *A. auricula-judae, B. adusta, Polyporaceae sp. (Irpex lacteus), M. scorodonius*, *T. albuminosus, P. ostreatus, T. versicolor, P. sapidus, and C. trogii* are indicated with blue arrows.

DyP1 then underwent a phylogenetic analysis with 241 DyP sequences from 88 fungal species belonging to 15 orders of this subphylum available at the JGI-DOE MycoCosm portal [38] and GenBank. Sequences within the resulting phylogram (Figure 2) can be sorted into the seven evolutionary clusters previously described by Linde et al. (2015a) [20]. Cluster I and III are the best defined and include most of the protein sequences. Cluster III mainly comprises sequences from Agaricales and Polyporales, with different enzymes characterized such as DyP4 from *P. ostreatus* [58], DyP1 from *T. versicolor* [30], and DyP from *Coriolopsis trogii* [59], and a hypothetical DyP cloned from *Ganoderma lucidum* [60] (blue arrows in Figure 2). Cluster I also includes Agaricales and Polyporales sequences, but here we can also find sequences from the orders Auriculariales, Sebacinales, and Geastrales, which are not represented in the rest of the clusters, and include eight characterized fungal DyPs from *B. adusta* [61], *Termitomyces albuminosus* (UniProtKB/Swiss-Prot Q8NKF3) [62], *A. auricula-judae* [28], *Mycetinis scorodonius* (two enzymes) [63,64], *Polyporaceae* sp. [65], *P. ostreatus* [58], and *Pleurotus sapidus* [66]. The sequence of DyP1 that we obtained from mangrove soils lies within Cluster I, flanked by Auriculariales and Sebacinales sequences, suggesting that the fungal species producing this enzyme most likely belongs to one of these two orders.

**Figure 3:**
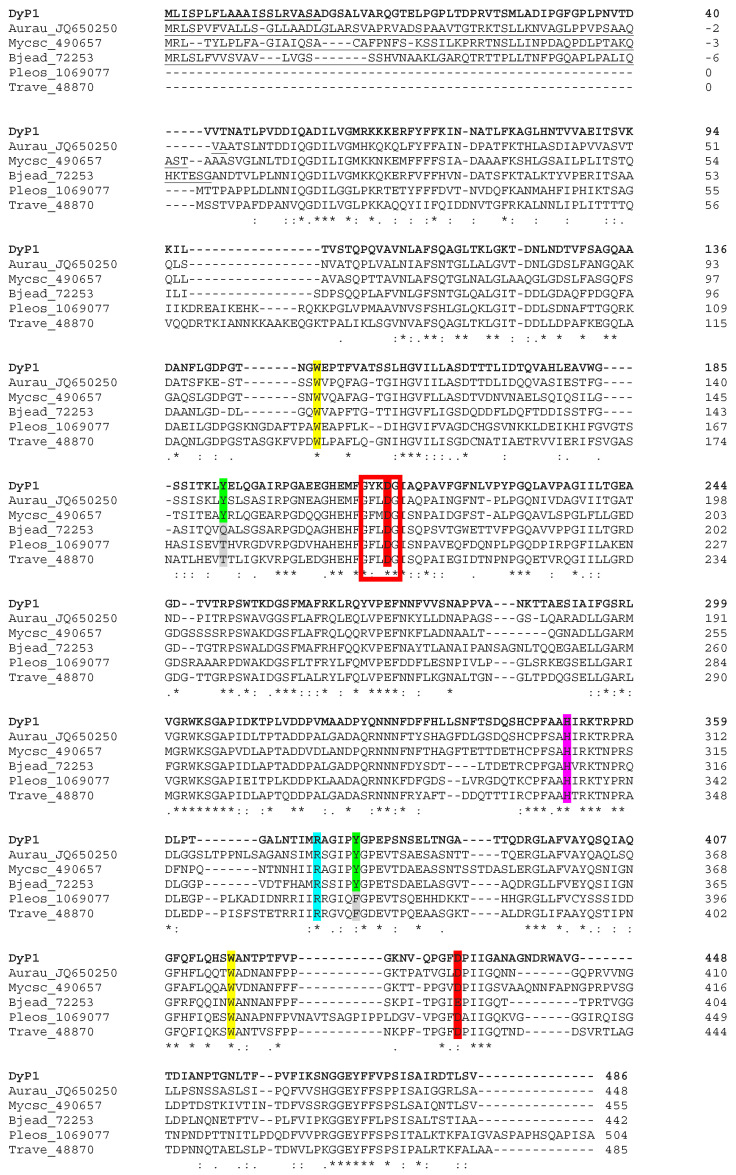
Alignment of DyP1 and five characterized DyPs from Cluster I (Aurau_JQ650250, Mycsc_490657, and Bjead_72253) and III (Pleos_1069077 and Trave_48870). Highlighted residues include (i) proximal histidine (magenta) and aspartate (red); (ii) distal-side arginine (cyan) and aspartate (red), the latter within the GXXDG signature motif (red box); and (iii) four solvent-exposed aromatic residues, corresponding to two conserved tryptophans (yellow), as well as two tyrosines (green) sometimes substituted by other amino acids (gray). Alignment was produced with Clustal2.1, and symbols below the sequences indicate full conservation of the same (asterisk) or equivalent residues (colon) and partial residue conservation (dot).

An alignment with five characterized fungal DyPs (Figure 3) representative of Clusters I and III confirmed that DyP1 presented the key amino acid residues characteristic of this peroxidase family, including (i) distal arginine and aspartate residues (Arg371 and Asp213), the latter forming part of the DyP signature motif GXXDG, necessary for enzyme activation by H_2_O_2_ [61], and (ii) the proximal histidine (His351) occupying the fifth coordination position of the heme iron, and a second aspartate (Asp433) [67]. Interestingly, some surface aromatic residues putatively involved in catalysis were also identified in the amino acid sequence of DyP1, as described in more detail below.

### 3.3. Structural Analysis

**Figure 4:**
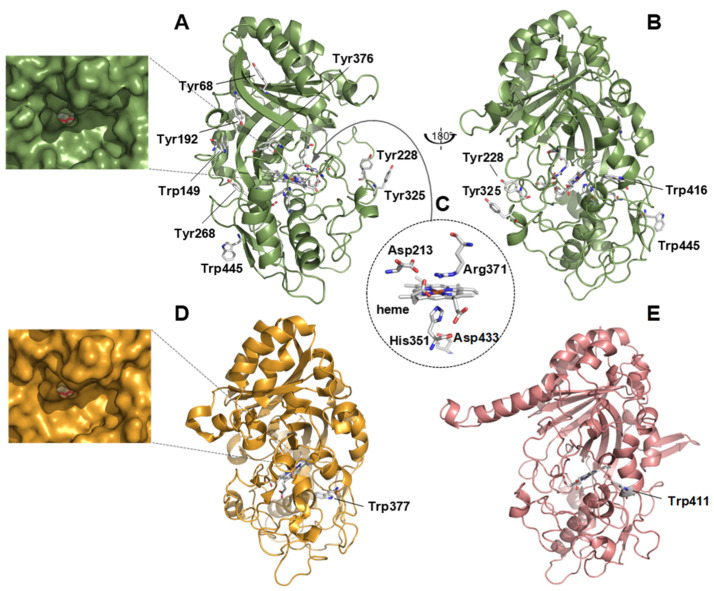
Homology model of DyP1 from mangrove soils, with a typical ferredoxin-like fold, showing the location of Trp and Tyr residues exposed to the solvent in two different orientations of the protein (**A**,**B**); and the heme environment amino acid residues Asp213, Arg371, His351, and Asp433 (as a structural detail in (**C**)). Trp416 in (**B**) is located at a position equivalent to that of the catalytic Trp377 in the *A. auricula-judae* DyP (*Aau*DyP) crystal structure (PDB: 4W7J) (**D**); and of Trp411 in a homology model of *T. versicolor* DyP (JGI 48870) (**E**). A detail of the heme access channel region in DyP1 and *Aau*DyP is also shown in A and D (the heme cofactor is depicted as red sticks at the bottom of the channel).

A DyP1 structural model was generated by homology modeling (Swiss Model). An analysis of this model revealed a ferredoxin-like fold with an internal heme cofactor accessible from the solvent through a narrow channel (Figure 4A,B). No significant differences were observed in size when the heme access channel was compared with that of the *A. auricula-judae* DyP (*Aau*DyP) (Figure 4D) where this enzyme could oxidize different substrates [20]. Different key residues identified in the amino acid sequence (Figure 3) are located at positions of the molecular architecture typical of a catalytically active enzyme (Figure 4C). The sequence includes six tryptophans and nine tyrosines with some of them (Tyr68, Trp149, Tyr192, Tyr228, Tyr268, Tyr325, Tyr376, Trp416, and Trp445) exposed to the solvent. The oxidation of bulky and high redox potential substrates at surface aromatic residues, via long-range electron transfer pathways to the heme, is known in ligninolytic peroxidases and DyPs [61,68]. Among the above aromatic residues, Trp149 and Trp416 are conserved in the five characterized DyPs shown in Figure 3. Trp416 occupies the same position of the catalytic Trp377 in the *A. auricula-judae* DyP [61] (Figure 4D) and of Trp411 (the only solvent-exposed aromatic residue) in the *T. versicolor* DyP (Figure 4E), pointing to a putative catalytic role of this residue in DyP1 (and also in *T. versicolor* DyP).

### 3.4. Heterologous Production and Purification of the Recombinant DyP1

After the transformation of *P. pastoris* with the recombinant vector pPICZα-A containing the DyP1 encoding cDNA, 48 transformants were selected for their resistance to zeocin and were then screened for the presence of DyP1 in the extracellular medium. The best transformant was selected based on the band intensity corresponding to the recombinant protein and visualized following SDS-PAGE (expected molecular weight for DyP1 was 55 kDa).

As dye-peroxidases are heme enzymes, the heme precursor hemin was added to *Pichia* cultures at two different concentrations (0.1 and 0.5 g L^−1^), to favor the production of functional holo-DyP1. SDS-PAGE analysis showed that the intensity of the band from the culture supplemented with 0.1 g L^−1^ hemin was higher than that of 0.5 g L^−1^. According to Zerva et al. [69], the presence of 0.1 g L^−1^ of hemin in the culture medium increases enzyme production more than 20-fold. With this approach, a soluble and active protein was produced, with a yield of about 290 mg of protein per liter of culture medium. The high production yield obtained in *P. pastoris* was much higher than that obtained with *E. coli* production with a few mg per liter, i.e., *Trametes versicolor Tv*DyP1 and *Pleurotus ostreatus* DyP isoforms *Pleos*DyP1 and *Pleos* DyP4 were produced with 1 mg/5 L and 6.3 to 11.7 mg/9 L of *E. coli* cultures, respectively [35,58].

The expected molecular weight for DyP1 was 55 kDa, but after purification, the protein ran on SDS-PAGE at about 70 kDa, with a 30% greater apparent molecular weight (Figure S1). This is due to the presence of N- and O-glycosylations, as already observed for other recombinant proteins produced in *P. pastoris* [70]. DyP1 was predicted to possess potential N-glycosylation at positions 23, 57, 67, 133, 163, 215, 374, and 546, as predicted via the N-GlyDE web server (http://bioapp.iis.sinica.edu.tw/N-GlyDE/, accessed on 19 April 2021). The best transformant was cultured in a larger volume (500 mL), and the recombinant protein was purified by affinity chromatography using an IMAC column. DyP1 was purified to homogeneity (Figure S1) from a culture containing 4682 mg of proteins with a recovery of 63.3 mg of DyP (Table 3). The recovery of the purification was 43.6%, with a purification factor of 32.4.

### 3.5. Catalytic Properties

Six different substrates, i.e., Mn^2+^, the anthraquinone dye RB19, the low redox-potential dye ABTS, together with the phenolic and non-phenolic aromatic compounds DMP and VA, were tested to evaluate substrate specificity of recombinant DyP1. The enzyme exhibited activity against ABTS and RB19 only (Table 4), with *K*_m_ values of 0.651 and 1.497 mM, respectively. No activity was found against DMP, Mn^2+^, or VA. Catalytic efficiency (*K*_cat_/*K*_m_) was estimated for the anthraquinone RB19 to 2.23 s^−1^ mM^−1^.

### 3.6. Enzyme Activity and Stability at Different pH and Temperature

**Figure 5:**
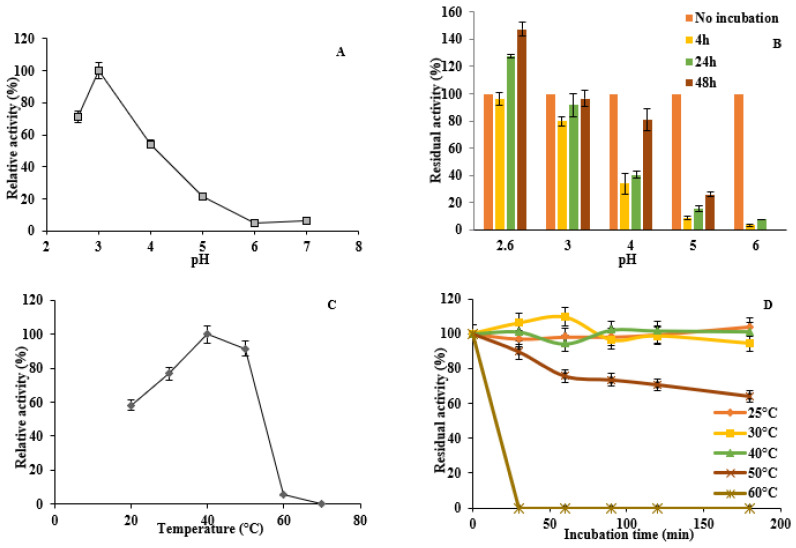
Effect of pH and temperature on enzyme activity and stability. (**A**) Optimal pH for the oxidation of ABTS (5 mM); (**B**) pH stability in the range pH 2.6–6 for 4, 24, and 48 h incubation; (**C**) optimal temperature for the oxidation of ABTS; and (**D**) temperature stability in the range 25–70 °C. Enzyme activity was measured in a 0.1 M citrate-phosphate buffer using ABTS (5 mM) as a reducing substrate and 0.25 mM H_2_O_2_ at 30 °C (and at pH 3.0 in (**C**,**D**). Activity values were calculated as a percentage of maximum activity (set to 100%) at optimum temperature and pH. Each data point (mean +/− standard deviation) is the result of triplicate experiments.

Recombinant DyP1 was active under acidic pH conditions with an optimum at pH 3 for ABTS in the pH range tested (Figure 5A). This value was 2.6 for RB19. At higher pH, the DyP1 activity substantially dropped when the pH was between 4 and 6, and almost no activity was found at pH 6.0. The pH stability of DyP1 was assessed by incubating the enzyme for 4, 24, and 48 h with ABTS in a pH range from 2.6 to 6. The enzyme turned out to be very stable throughout the range of pH and the activity increased with time, with a marked activation at pH 4 and 5 (Figure 5B). However, the enzyme lost its activity after 48 h of incubation at pH 6.

The optimum temperature for the enzyme activity against ABTS was 40 °C. However, the enzyme was less active in the ranges 20–30 and 50–55 °C (60–80%, respectively, compared to its activity at 40 °C), and nearly lost its activity at 60 °C (Figure 5C). The thermal stability of DyP1 was examined by testing activity towards ABTS after heat treatment of the enzyme at different temperatures and for various incubation times, ranging from 30 to 180 min. The enzyme was stable at temperatures ranging from 30 to 50 °C, retaining about 60% of initial activity after 180 min of incubation at 50 °C. However, at 60 °C and above, no activity remained after 30 min of incubation (Figure 5D).

### 3.7. Decolorization of Industrial Dyes

Because of its potential for dye decolorization, DyP1 activity was tested on five more industrially relevant dyes, belonging to five different chemical classes: acidic, basic, reactive, vat, and disperse dyes. As shown in Table 5, DyP1 was highly active on Reactive Black 5 (RB5) dye (32.3% of decolorization) and to a lesser extent on Acid Black (AB) and Disperse Blue 79 (DB). By contrast, *Tv*DyP1 from *T. versicolor* (GenBank accession numbers 19415892) was active only against AB, though with 75% of decolorization.

### 3.8. Effect of Hydrogen Peroxide on DyP1 Activity

**Figure 6:**
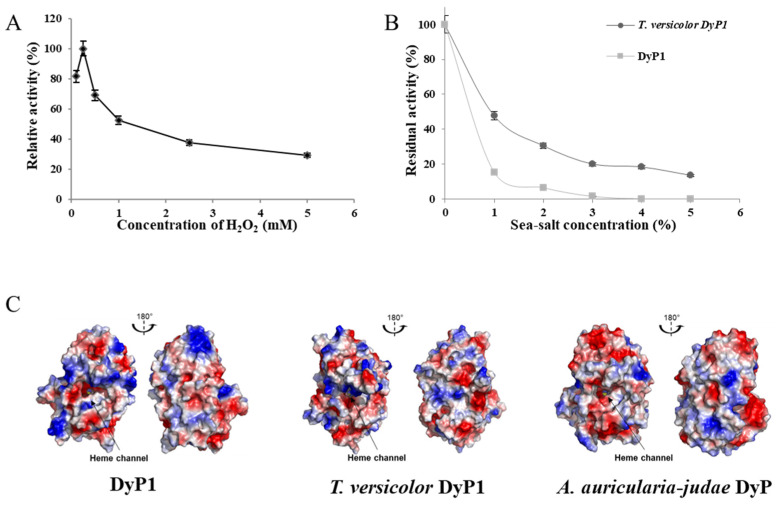
Effect of hydrogen peroxide and sea salt on DyP1 activity and surface charges of three different fungal DyPs. (**A**) The optimal concentration of hydrogen peroxide in standard conditions, using ABTS (5 mM) as a substrate. (**B**) Effect of sea salt addition on recombinant DyP1 and *T. versicolor* DyP (*Tv*DyP1) as a control. Relative enzymatic activity recorded as in (**A**). Each data point (mean +/− standard deviation) is the result of triplicate experiments. (**C**) Surface charge plots (negative and positive charges in red and blue, respectively) from homology models of DyP1 and *Tv*DyP1 (GenBank accession numbers 19415892) and from the experimental three-dimensional structure of *Aau*DyP from *A. auricula*-*judae* (PDB: 4W7J). The heme cofactor, deeply buried within the active site, is hardly visible (yellow spheres). Surface potentials were calculated using the vacuum electrostatics function of the PyMOL molecular graphics system (Schrödinger, New York, NY, USA).

Although hydrogen peroxide is the electron acceptor of peroxidases, these enzymes are known to lose activity in the presence of H_2_O_2_, through a mechanism known as suicide inactivation [71]. The optimum concentration of H_2_O_2_ was determined by incubating the reaction mixture with different concentrations of H_2_O_2_, ranging from 0.1 to 5 mM, and the highest DyP1 activity was recorded for 0.25 mM H_2_O_2_ (Figure 6A). Above this concentration, DyP1 residual activity decreased gradually, up to 30% at 5.0 mM H_2_O_2_.

### 3.9. Influence of Sea Salt on DyP1 Activity and Surface Charge of the Recombinant DyP1

Recombinant DyP1, identified in mangrove sediments, was compared with *Tv*DyP1, active in terrestrial environments, to gain insights into DyP1 adaptation to saline conditions, as found in marine environments. As we can see in the results presented in Figure 6, the activity of the purified DyP1 was affected by sea salt addition (Figure 6B). From 1% of sea salt addition, the enzyme retained less than 20% of its initial activity. At 3% of sea salt, the recombinant DyP1 activity was severely affected by sea salt, even more than its terrestrial counterpart, *Tv*DyP1.

Three-dimensional models of recombinant DyP1 and *Tv*DyP1 were generated, and the overall surface charges were compared with *Aau*Dyp (Figure 5C). The three enzymes showed a well-balanced ratio of negative to positive surface charges. In line with these results, analysis of the enzyme primary structure showed that the ratio of negatively charged (D + E) over positively charged (R + K) amino acids was about 1.19, 1.16, and 1.24 for the recombinant DyP1, *Tv*DyP1, and *Aau*DyP, respectively. By contrast, a lytic-polysaccharide monooxygenase cloned from the mangrove fungus, *Pestalotiopsis* sp NCi6 (*Ps*LPMOA), and a laccase obtained from the marine-derived *Pestalotiopsis* sp KF079 possessed higher (D + E)/ (R + K) ratios of 4.8 and 3.95, respectively.

## 4. Discussion

The mangrove ecosystem accounts for a large production of lignocellulosic biomass and greatly contributes to carbon sequestration on the planetary scale [72]. Fungi are colonizers of mangrove forests, and the representative species form a large and diversified ecological group, playing a central role in the degradation of lignocellulosic sedimentary organic matter [73]. Lignocellulose biomass degradation is based on the secretion of a broad variety of enzymes that have different, complementary catalytic activities, including cellulases, hemicellulases, and lignin-modifying enzymes [74]. In this study, we focused on DyPs as model lignin-modifying enzymes, because enzymes from this family are present in Ascomycota and Basidiomycota, and both Phyla were identified in New Caledonian mangrove sediments [3]. These enzymes have been suggested to contribute to the degradation of phenolic residues and the modification of lignin-derived soil organic matter [61].

The diversity of expressed genes encoding fungal DyPs was investigated in both surface and buried mangrove sediments underneath *A. marina* and *R. stylosa* trees, and during the wet (March) and the dry (November) seasons. Compared to soils from terrestrial forests, which can harbor a high diversity of expressed lignolytic genes (e.g., from 82 to 253 DyP OFUs [75]), mangrove sediments showed much narrower diversity, with only 25 DyP OFUs retrieved, possibly the result of physical–chemical gradients associated with mangroves sediments that are inimical to fungal ligninolytic enzyme activities (e.g., low oxygen and nitrogen contents [76]). DyP diversity was also strongly affected by the season, as already observed for the fungal community composition [35]. A systematic decrease in DyP diversity was observed in surface layers during the dry season (November). In previous studies, wet seasons appeared favorable to microbial colonization in mangrove ecosystems, particularly for fungi [73], as salinity and temperature decrease and increase, respectively [77,78]. Moreover, the water availability is an important factor regulating fungal activity in mangrove sediments [79]. However, in our study, DyP diversity was lower in the wet season, except for surface layer sediments collected during the wet season beneath *R. stylosa*. The impact of tree species on DyP composition is consistent with previous work conducted in forest ecosystems, showing that tree species and more particularly species-generated soil C/N ratio are the most important factors driving functional gene distribution [75]. However, in New Caledonian mangrove habitats, salinity is also an important factor. It shapes tree distribution, impacting directly or indirectly on the taxonomic and functional diversity of sediment fungal communities [80]. Random high-throughput (Hiseq) sequencing of environmental cDNA after gene capture by hybridization enabled us to identify the full-length sequence of the most abundant fungal OFU in mangrove sediments, DyP1, whose sequence was successfully cloned and expressed in *P. pastoris*.

The origin of DyP1 was inferred from phylogenetic analysis, and a biochemical characterization was conducted to determine its physical–chemical properties, kinetic parameters, and potential for biotechnological applications, such as dye decolorization. Phylogenetic analysis revealed a sequence similarity from (47% to 59%) of the recombinant DyP1 with those of DyPs from four species of the orders Auriculariales and Sebacinales (Basidiomycota, Agaricomycotina), including *E. glandulosa*, *A. delicata,* *P. indica*, and *S. vermifera*. This result is in agreement with our previous finding that Basidiomycota was mainly represented by Agaricomyceta [3]. Although Ascomycota largely dominated the analyzed mangrove sediments (73.8–94.5% of total ITS sequences), Basidiomycota represented 5.3–26.2% of total ITS sequences [3]. Interestingly, Agaricomycotina represented 5.3–18.3% of total ITS sequences, i.e., most of the Basidiomycete sequences, and operational taxonomic units (OTUs) affiliated to the genus *Exidia* were detected in mangrove sediments.

The structural model of DyP1 highlighted the presence of the heme cofactor and a ferredoxin-like fold forming the CDE superfamily. Asp 213 and Arg 371 were identified showing the conserved motif GXXDG residues and their contribution in the heterolytic cleavage of H_2_O_2_ to activate the enzyme. This result was similar to *Aau*DyP where Aspartate and Arginine were located in positions 168 and 332, respectively [61], and to *P. sapidus* DyP with Asp174 and Arg338 [66]. Additionally, the fifth ligand of the heme iron of DyP1, histidine 351, was detected with an aspartate at position 433 forming a hydrogen bond similar to the recombinant PsaDyP (His 317 and Asp 401) and *Aau*DyP (His304 and Asp395). However, in some cases, the aspartate residue was substituted by a glutamic acid [61,66]. The homology modeling admits the catalytic activity of the Trp 416 of DyP1, which was tendentially like *A. auricularia-judae* (Trp 377) and *T. versicolor* DyP (Trp 411) [30,61]. To oxidize a large number of substrates, DyP1 shows an aromatic surface with tryptophane and tyrosine residues. This result was in line with PsaDyP, where Tyr343 and Trp383 were conserved solvent-exposed residues, and with *Pleurotus eryngii* VP, where Trp164 plays a key role in the direct electron transfer [66,68].

Among the five substrates tested, including Mn^2+^, recombinant DyP1 showed activity on ABTS and RB19 only, with higher affinity for the former (*K*_m_, 651.39 µM) than for the latter (*K*_m_, 1497.07 µM). Affinities for ABTS and RB19 were higher for *Tv*DyP1 (*K_m_*, 292 and 37.8 µM, respectively) and *Aau*DyP (*K*_m_, 283 and 6.5 µM respectively). For *P. ostreatus* DyPs, *Pleos* yP1/ *Pleos*DyP4 *K*_m_ values were 780 and 787 µM for ABTS, and 45 and 82 µM for RB19, respectively [58]. *I. lacteus* DyP also showed higher affinity to ABTS (*K*_m_ 28 µM) than to RB19 (*K*_m_ 13 µM) [65]. Catalytic efficiency of DyP1 was 4–5 times higher for RB19 *(k*_cat_/*K*_m_, 2.23 s^−1^ mM^−1^) than for ABTS (*k*_cat_/*K*_m_, 0.49 s^−1^ mM^−1^), whereas catalytic efficiency on ABTS was 3, 2, and 0.7 times higher than catalytic efficiency on RB19, for *Tv*DyP1, *Pleos*DyP1/ *Pleos*DyP4, and *I. lacteus* DyP, respectively [30,58,65]. In conclusion, although the catalytic efficiencies of recombinant DyP1 were lower than for the above characterized DyPs, they were still in the range of what was found for DyPs isolated from terrestrial environments.

The structural homology model obtained for DyP1 presents both the heme cofactor and the ferredoxin-like fold characteristic of the CDE superfamily [23]. A detailed analysis revealed all the elements characterizing a catalytically active DyP. Thus, Asp213 and Arg371 located over the heme plane (at the so-called distal side) are expected to contribute to the enzyme activation by H_2_O_2_ as previously demonstrated for distal aspartate and arginine in *Aau*DyP [20]. Similarly, the key residues located below the heme plane (the so-called proximal side) are conserved when compared with other DyPs. In this region, His351 therefore acts as the fifth ligand of the heme iron, and the interaction between this histidine and the neighboring Asp433 may be responsible for the redox potential of the enzyme, as suggested by Linde et al. [61]. Concerning the putative catalytic sites for substrate oxidation, the heme access channel and the surface Trp416, which occupies a position equivalent to that of the catalytic Trp377 in *Aau*DyP, are the main candidates. The former has been suggested to be the low-efficiency site for ABTS and RB9 oxidation, while the solvent-exposed Trp377 has been demonstrated to be the high-efficiency site for these two substrates in the *A. auricula-judae* enzyme [20]. Unlike this enzyme, DyP1 presents a set of kinetic constants for these two substrates, indicating that only one of these two sites is active, although other alternative sites cannot be ruled out. Among them, we found other solvent-exposed tryptophan and tyrosine residues that could also act as catalytic residues instead of Trp377. They could be activated by long-range electron transfer pathways in a similar way as described for Trp377 in *Aau*DyP [61] and for different Trp and Tyr residues in ligninolytic peroxidases [81,82,83]. Directed mutagenesis studies of these residues and at the heme access channel are necessary to definitively identify the catalytic site of this enzyme.

Enzymatic activity on ABTS of purified recombinant DyP1 was optimal at pH 3, similar to what was reported for DyPs from *I. lacteus* [65], *T. versicolor* [30], *P. sapidus* [66], *A. auricula-judae* [29], and *Pleurotus ostreatus* [58]. Recombinant DyP1 is stable at acid pH (2.6–5.0), with activity increase for longer incubations at pH 4.0 and 5.0. At pH 6.0, enzyme activation was observed for 24 h, whereas after a longer incubation time, activity was completely abolished. For *Tv*DyP, *P* *Pleos*DyP1/*Pleos*DyP4, and *I. lacteus* DyP, stability was similar in the same pH range, but no activation effect at pH 4.0 and 5.0 was observed [30,58,65]. The effect of temperature on DyP1 activity and stability was measured at different temperature points ranging from 30 to 70 °C. Recombinant DyP1 showed optimal activity at 40 °C and retained 60–80% residual activity after 180 min incubation at temperatures in the range of 30–50 °C. For corresponding temperatures, *Tv*DyP1 retained only 5–35% activity, and *Pleos*DyP1 was inactive, although *Pleos*-DyP4 maintained 100% activity even in the range of 60–70 °C [30,58]. Finally, *P. sapid*us DyP (*Psa*DyP) was active in the temperature range of 15–30 °C but had already lost 50% activity after 5 min incubation at 50 °C [66]. Although H_2_O_2_ is a peroxidase co-substrate, it is also known to inactivate enzyme activity above a critical, enzyme-specific concentration. Optimal activity for recombinant DyP1 was recorded at 0.25 mM H_2_O_2_. This value lies within the range of values determined for other DyPs: 0.125 mM for *Psa*DyP, 0.5 mM for *Tv*DyP1, and 0.4 to 0.8 mM for *I. lacteus* DyP [30,65,66].

To assess the potential of recombinant DyP1 as a biocatalyst for applications in white biotechnology, we tested its capacity to decolorize the industrial dyes already tested for *Tv*DyP1 [30]. DyP1 was active on three dyes (AB, RB5, and DB79), corresponding to different chemical classes (acidic, reagent, and disperse dyes), whereas *Tv*DyP1 was only active on AB, though with a higher efficiency (75% decolorization). DyP1 instead showed higher substrate versatility, suggesting an original technological potential for large-spectrum dye bleaching, and showed the best efficiency on RB5 (32% decolorization). In several studies, RB5 is reported as a well-known recalcitrant azo dye, with a rigid aromatic molecule that is difficult to degrade. This compound is dangerous, carcinogenic, and toxic to humans and the environment [84]. In previous studies, RB5 was decolorized by various fungal strains, such as *P. eryngii* F032 (94% decolorization) [85], *Geotrichum candidum* Dec 1 (94%) [86], *Cerrena sp.* WICC F39 (86%) [87], *Funalia trogii* (95%) [88], and *Trametes gibossa* WRF3 (82%) [84]. These results open a field of application for the recombinant DyP1 that needs to be further developed, for instance by immobilizing the enzyme to improve its efficiency [89].

To complete enzyme characterization, and as the recombinant DyP1 was obtained from a marine environment (mangrove of New Caledonia), we tested the behavior of DyP1 in saline conditions. Despite its marine origins, recombinant DyP1 was similarly affected by sea salt, compared with terrestrial-derived *Tv*DyP1. By contrast, marine *Phlebia* sp. MnP showed four times higher activity when the culture medium was supplemented with 3% of sea salt [15]. Furthermore, two laccases recently cloned from the marine-derived fungus *Pestalotiopsis* sp. KF079, isolated from the Baltic sea mudflats, were strongly activated by up to 360% of their initial activity in the presence of 5% (*w/v*) sea salt [33]. We also demonstrated that lytic polysaccharide monooxygenases from the mangrove fungus *Pestalotiopsis* sp. NCi6 (*Ps*LPMOA) remained active even at 6.0% (*w/v*) sea salt [90].

Salt-adapted enzymes originating from marine environments are generally characterized by highly negative surface charges thought to contribute to protein stability and activity in extreme osmolytic conditions [91,92,93]. In our results, the recombinant DyP1 had a low (D + E)/(R + K) ratio of 1.19, which is the same as for the terrestrial-derived *Tv*DyP1 (1.16) and *Aau*DyP (1.24). In line with these results, this ratio for the laccases *Mt*Lac from the terrestrial *Myceliophthora thermophila* was 1.55, and for *Ps*Lac1 from the marine-derived *Pestalotiopsis* sp. and *Sl*Lac2 from *S. lucomagnoense* 1.55 and 1.2, respectively, despite their marine origin [14,33]. By contrast, the laccase *Ps*Lac2 and *Ps*LPMOA enzymes isolated from *Pestalotiopsis sp.* had a four-times higher recurrence of negatively (D + E) than positively charged (R + K) amino acids [33,90]. This observation was supported by comparing the homology-guided three-dimensional models generated for two DyPs revealing a well-balanced surface charge distribution, while for the *Ps*Lac2 and *Ps*LPMOA enzymes highly negative residues were exposed at the surface [33,92]. We can thus conclude that the surface charge distribution of mangrove-derived DyP1 is reminiscent of those of terrestrial DyPs. Mangrove habitats are detritus-based ecosystems, colonized by a large community of terrestrial saprotrophic fungi [73]. Future experiments will be needed to compare the structural organization of the mangrove soil at the taxonomic and functional levels. To achieve this goal, it will be pivotal to clone and characterize other DyPs identified in the mangrove soil samples and to study DyP biodiversity as a function of depth, vegetation, and season. Similar studies should be also conducted on other lignocellulose-degrading enzymes to validate this approach and corroborate these results.

## 5. Conclusions

Mangrove habitats are rich ecosystems, extremely diverse because of the combination of highly variable environmental gradients related to salinity, temperature, humidity, depth, tree species, and many more variables. This results in a rich biodiversity and diverse microbial distribution, constituting as such a very original model environment to characterize. In the present work, gene capture by hybridization combined with high-throughput sequencing allowed the detection of fungal functional cDNAs encoding DyPs, whose expressions were lower than the genes identified in conventional forest soils, and the discovery of novel biocatalysts. We also expressed and characterized the most frequently encountered and abundant DyP from the explored mangrove. We conclude that the newly discovered DyP1 is expressed from a fungal species within the genus *Exidia* and that the enzyme has biochemical properties close to their terrestrial isoforms, although likely endowed with greater substrate versatility.

## Figures and Tables

**Table 1:** Effect of environmental factors on the composition of expressed fungal genes encoding dye-decolorizing peroxidases (DyPs).

	Df	F	P	R^2^
Variable				
Tree species	1	3.011	0.032	0.062
Sediment depth	1	3.453	0.021	0.072
Season	1	8.579	5.8 × 10^−5^	0.178
Interaction				
Tree × Depth	1	6.789	7.1 × 10^−4^	0.141
Tree × Season	1	2.462	0.062	0.051
Depth ×Season	1	2.926	0.036	0.061
Tree × Depth × Season	1	4.946	0.005	0.102
Residuals	16			

The differences between groups were tested using PERMANOVA analysis on Bray–Curtis dissimilarity matrices. Abbreviations: Df: degrees of freedom; *F*: *F*-test statistic.

**Table 2:** DyPs deposited from the UniProtKB/Swiss-Prot database presenting the highest amino acid sequence identities (47–59%) with DyP1 from mangrove soils (the number of residue pairs considered for each comparison is shown in brackets). The DyP entry names of 12 enzymes are indicated together with the fungal species they belong to, from the orders Auriculariales (*Exidia glandulosa* and *A. auricula*-*judae*), Sebacinales (*Piriformospora indica* and *Serendipita vermifera*), and Geastrales (*Sphaerobolus stellatus*).

	DyP1
*E. glandulosa* DyP A0A165BX62	59% (459)
*P. indica* DyP G4TL25	57% (460)
E. glandulosa DyP A0A165FCE7	56% (460)
*E. glandulosa* DyP A0A165G2C1	54% (498)
*E. glandulosa* DyP A0A165GZG2	54% (469)
*S. stellatus* DyP A0A0C9U2H4	54% (465)
*S. vermifera* DyP A0A0C3B0S6	53% (460)
*E. glandulosa* DyP A0A166ARP7	52% (505)
*A. auricula-judae* DyPI2DBY1	51% (505)
*S. stellatus* DyP A0A0C9UT91	51% (505)
*S. stellatus* DyP A0A0C9VF44	49% (472)
*S. stellatus* DyP A0A0C9VPJ8	47% (506)

**Table 3:** Purification for the recombinant DyP1 produced in *Pichia pastoris* *X33*. IMAC: immobilized metal affinity chromatography.

Purification Step	Volume (mL)	Total Activity (U mL^−1^)	Protein (mg)	Specific Activity (U mg^−1^)	Yield (%)	Purification (Fold)
Culture medium	500	376	4682	0.08	100	1
IMAC	25	164	63.3	2.59	43.6	32.4

**Table 4:** Kinetic constants of the recombinant DyP1 produced in *P. pastoris*. The pH column indicates either the tested pH range or the determined optimal reaction pH.

Substrate	Parameters			
	*K*_m_ (mM)	*k*_cat_ (s^−1^)	*k*_cat_/*K*_m_ (s^−1^ mM^−1^)	pH
ABTS	0.651 +/− 0.081	0.322	0.49	3
RB19	1.497 +/− 0.878	3.34	2.23	2.6
DMP	0	0	0	2.6–7
Mn^2+^	0	0	0	2.6–6
VA	0	0	0	2.6–7

**Table 5:** Decolorization of industrial dyes by the recombinant DyP ^a^.

Dye	DyP1	*Tv*DyP1
AB	18.8 +/− 0.008	75.0 +/− 0.007
BB	−	−
RB5	32.3 +/− 0.009	−
DB79	5.2 +/− 0.005	−
VG	−	−

^a^ Decolorization was determined after 1 h of incubation in citrate–phosphate buffer (100 mM, pH 3) and 0.25 mM of H_2_O_2_ at 37 °C. Symbols: −: no decolorization. Each data point (mean +/− standard deviation) is the result of triplicate experiments.

## Supplementary Materials

The following are available online at https://www.mdpi.com/article/10.3390/jof7050321/s1. Table S1: List of tagged primers used to amplify expressed genes encoding fungal Dye-decolorizing Peroxidases (DyPs); Table S2: Normalized dataset where the 781,344 fungal DyP sequences are distributed among the 25 different Operational Functional Units (OFUs) within the sediment samples; Table S3: Mean number of fungal DyP OFUs (Operational Function Units) detected and diversity indices calculated for oxic and anoxic sediment fractions collected during the wet (March) and dry (November) seasons within two mangrove pristine areas (*Avicennia marina* and *Rhizophora stylosa*); Figure S1: title: SDS-PAGE of the two-step purification for the recombinant DyP1 produced in *Pichia pastoris*. Lanes: 1, culture medium fraction; 2, purified DyP1 obtained after IMAC chromatography and M, protein molecular mass markers.
