# Supplementary material for: Exploring the Diversity of Fungal DyPs in Mangrove Soils to Produce and Characterize Novel Biocatalysts
Source: J Fungi (Basel). 2021 Apr 21;7(5):321. doi: 10.3390/jof7050321 (PMC8143184; doi:10.3390/jof7050321)
Supplement: Supplementary file 1 [file jof-07-00321-s001.zip › 8.jof-1179052-supplementary/9.Figure S1.pdf]

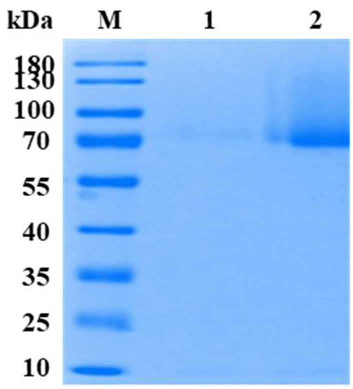

Figure S1. SDS-PAGE of the two-step purification for the recombinant DyP1 produced in *Pichia pastoris*. Lanes: 1, culture medium fraction; 2, purified DyP1 obtained after IMAC chromatography and M, protein molecular mass markers.
